# Supplementary material for: Male Knock-in Mice Expressing an Arachidonic Acid Lipoxygenase 15B (Alox15B) with Humanized Reaction Specificity Are Prematurely Growth Arrested When Aging
Source: Biomedicines. 2022 Jun 10;10(6):1379. doi: 10.3390/biomedicines10061379 (PMC9220125; doi:10.3390/biomedicines10061379)
Supplement: Supplementary file 1 [file biomedicines-10-01379-s001.zip › biomedicines-1745682-supplementary.pdf]

Supplemental information to the paper

Male knock-in mice expressing an arachidonic acid lipoxygenase 15b (Alox15b) with humanized reaction specificity are prematurely growth arrested when aging

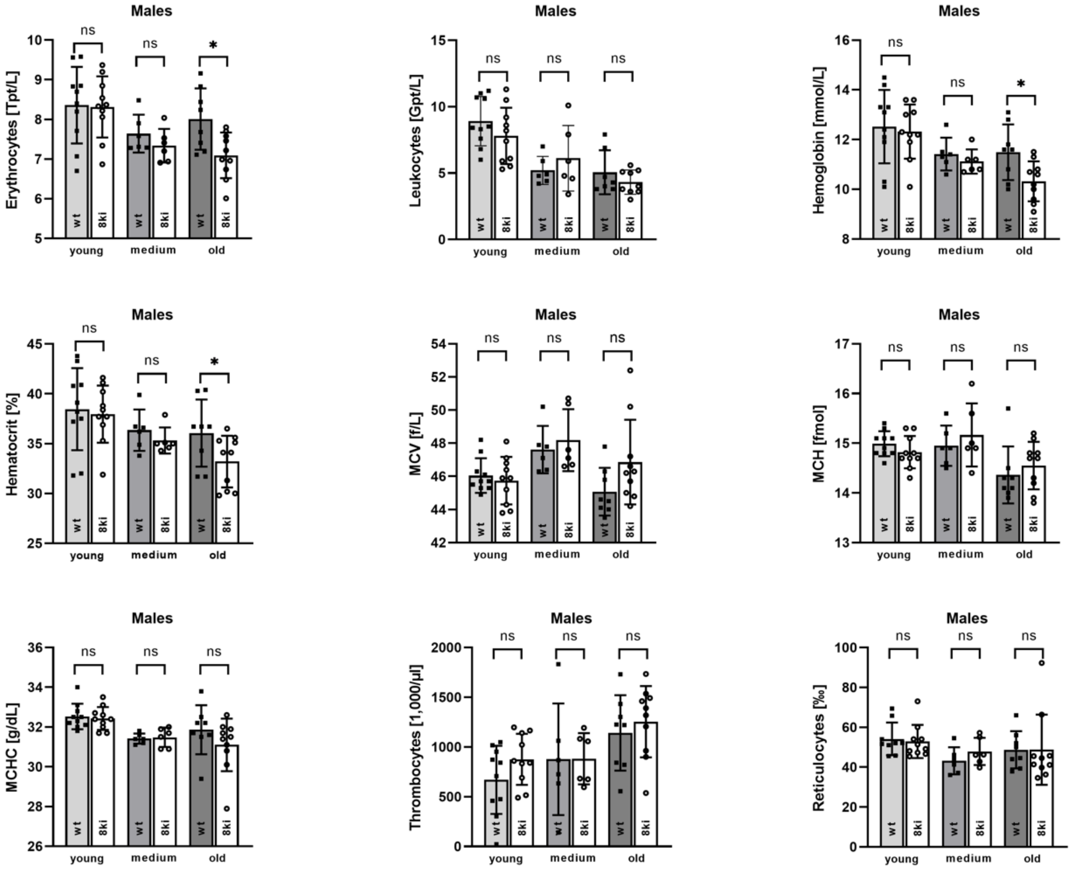

Figure S1. Basic hematological parameters of male Alox15-KI mice and outbred wildtype control animals of different age categories. Alox15b-KI mice and outbred wildtype controls of either sex were classified in three age categories (young mice, 10-20weeks; middle-aged mice, 30-40 weeks; old mice, 70-80 weeks, n≥5 for each age group). After sacrificing the animals by cervical dislocation under anesthesia EDTA blood was removed by heart puncture. The basic hematological parameters were determined by Institut für Veterinärmedizinische Diagnostik GmbH (Berlin, Germany).

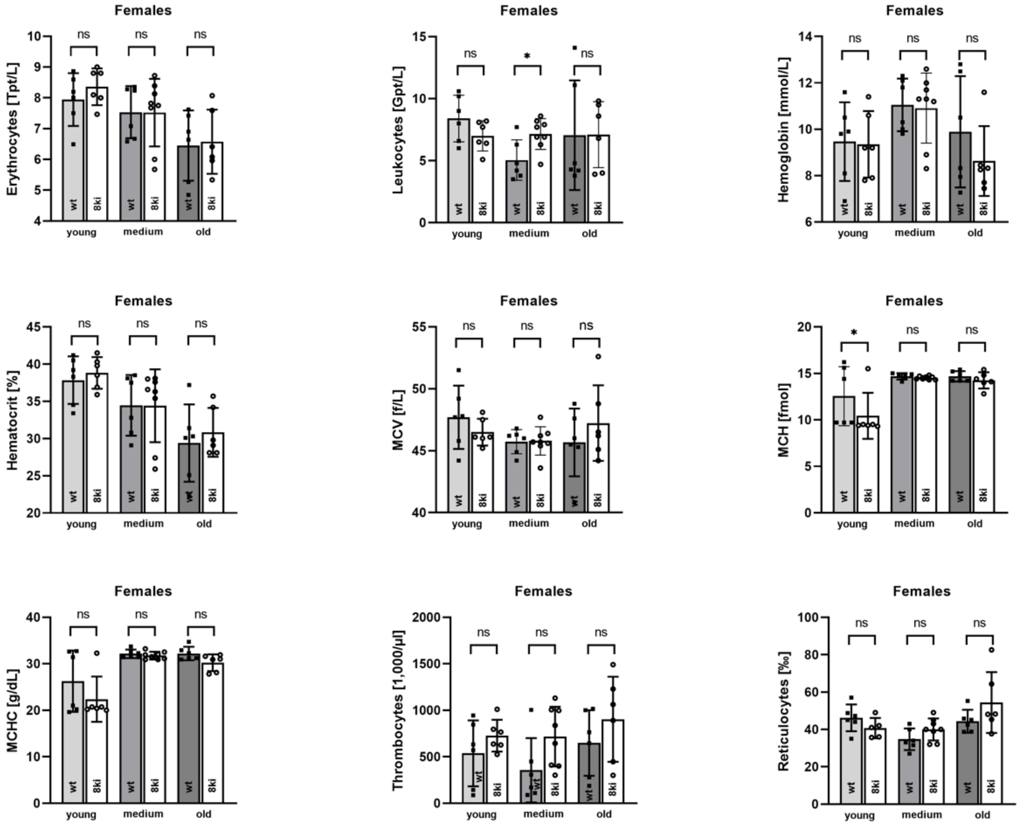

Figure S2. Basic hematological parameters of female Alox15-KI mice and outbred wildtype control animals of different age categories. Alox15b-KI mice and outbred wildtype controls of either sex were classified in three age categories (young mice, 10-20 weeks; middle-aged mice, 30-40 weeks; old mice, 70-80 weeks, n≥5 for each age-group). After sacrificing the animals by cervical dislocation under anesthesia EDTA blood was removed by heart puncture. The basic hematological parameters were determined by Institut für Veterinärmedizinische Diagnostik GmbH (Berlin, Germany).

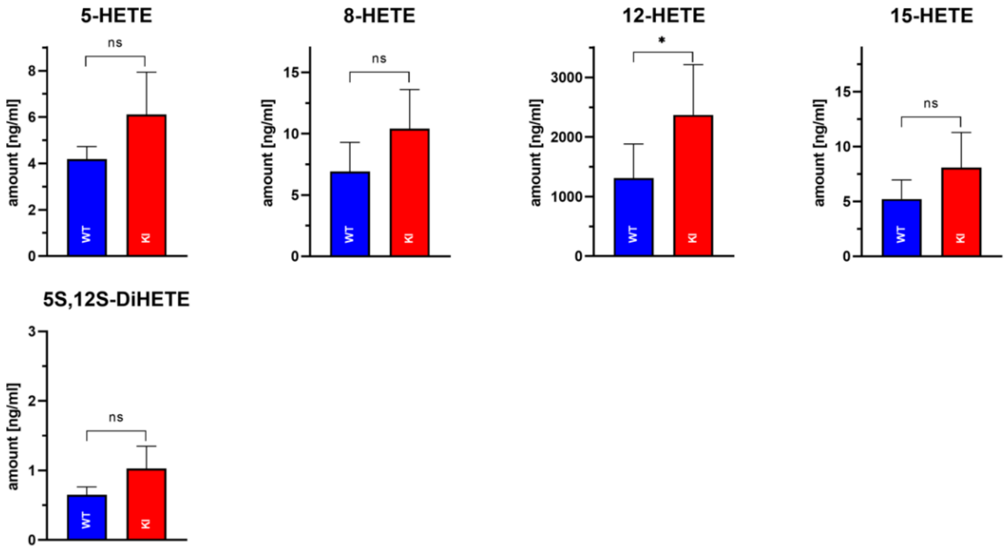

Figure S3. Quantification of the major free oxygenated arachidonic acid metabolites in the blood plasma of Alox15b-KI mice and outbred wildtype controls. The lipids were extracted from the blood plasma of Alox15b-KI mice and outbred wildtype controls (n=5 each) and the specified free oxylipins were analyzed by LC-MS (see Materials and Methods).

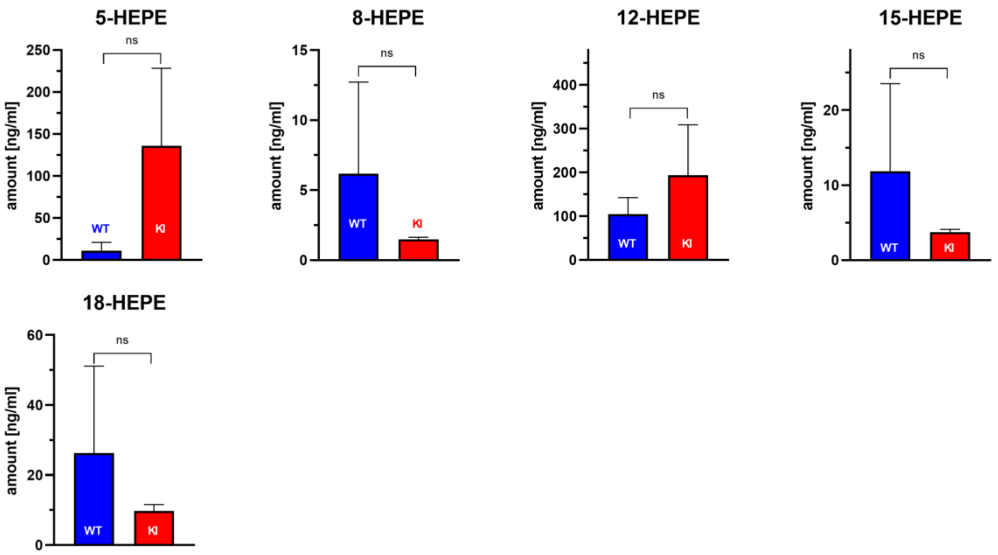

Figure S4. Quantification of the major free oxygenated 5,8,11,14,17-eicosapentaenoic acid metabolites in the blood plasma of Alox15b-KI mice and outbred wildtype controls. The lipids were extracted from the blood plasma of Alox15b-KI mice and outbred wildtype controls (n=5 each) and the specified free oxylipins were analyzed by LC-MS (see Materials and Methods).

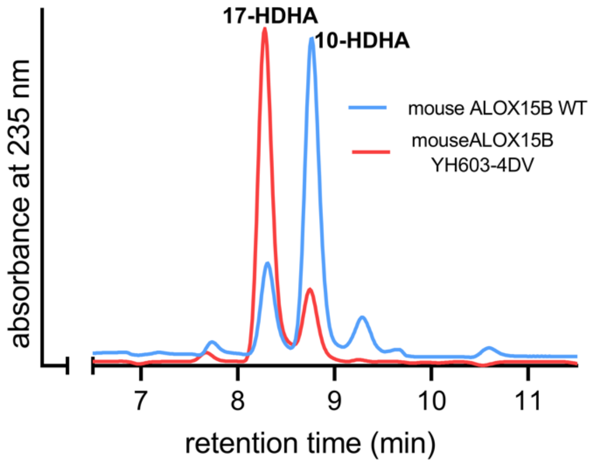

Figure S5. RP-HPLC analysis of the major conjugated dienes formed from 4,7,10,13,16,19-docosahexaenoic acid (DHA) by recombinant mouse Alox15b and its Tyr603Asp+His604Val double mutant exhibiting a humanized reaction specificity with arachidonic acid as substrate. Wildtype recombinant mouse Alox15b and its Tyr603Asp+His604Val double mutant were expressed as N-terminal his-tag fusion proteins in *E. coli* (see Materials and Methods) and the bacterial lysis supernatants were used as enzyme source. Aliquots of the lysis supernatant were incubated in PBS with 100 μM of DHA. The oxygenation products were prepared and analyzed by RP-HPLC (see Materials and Methods). The retention times of authentic standards are indicated by the arrows above the traces and the MS spectra of the major conjugated dienes (data not shown) confirmed their chemical structures.

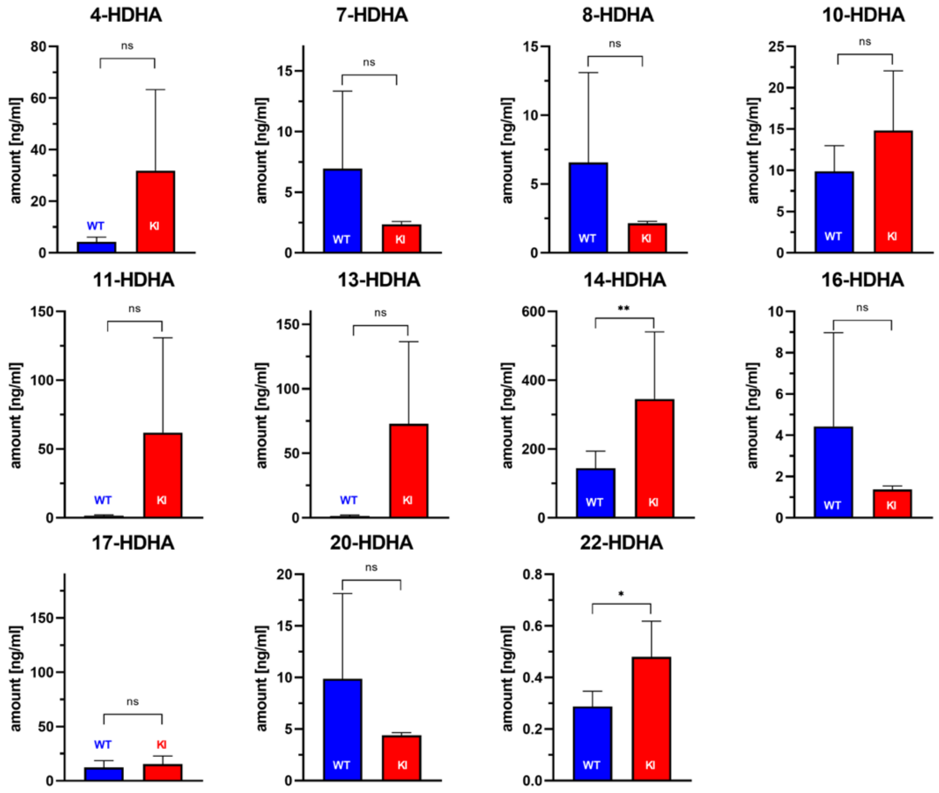

Figure S6. Quantification of the major free oxygenated 4,7,10,13,16,19-docosahexaenoic acid metabolites in the blood plasma of Alox15b-KI mice and outbred wildtype controls. The lipids were extracted from the blood plasma of Alox15b-KI mice and outbred wildtype controls (n=5 each) and the specified free oxylipins were analyzed by LC-MS (see Materials and Methods).

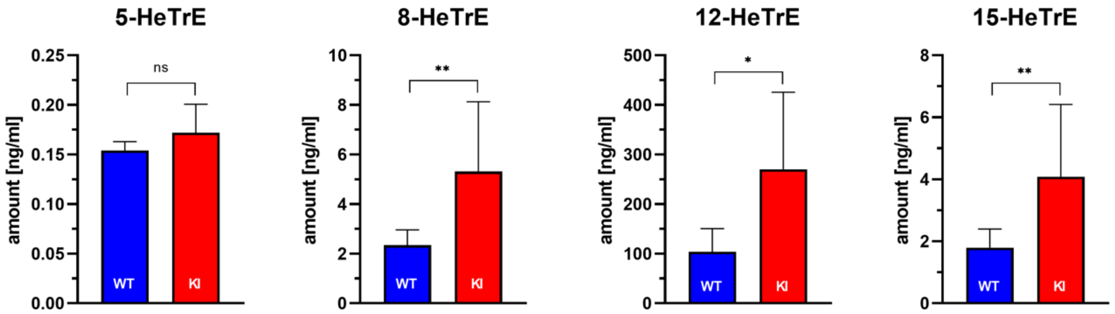

Figure S7. Quantification of the major free oxygenated metabolites of 8,11,14-eicosatrienoic acid in the blood plasma of Alox15b-KI mice and outbred wildtype controls. The lipids were extracted from the blood plasma of Alox15b-KI mice and outbred wildtype controls (n=5 each) and the specified free oxylipins were analyzed by LC-MS (see Materials and Methods).

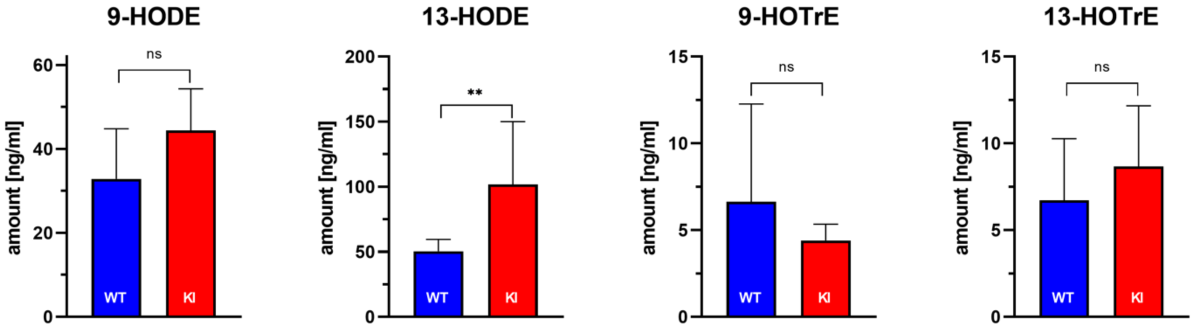

Figure S8. Quantification of the major free oxygenated linoleic acid (HODE-isomers) and alpha-linolenic acid metabolites (HOTrE-isomers) in the blood plasma of Alox15b-KI mice and outbred wildtype controls. The lipids were extracted from the blood plasma of Alox15b-KI mice and outbred wildtype controls (n=5 each) and the specified free oxylipins were analyzed by LC-MS (see Materials and Methods).

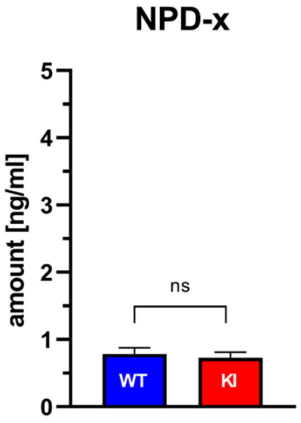

Figure S9. Quantification of free NPx in the blood plasma of Alox15b-KI mice and outbred wildtype controls. The lipids were extracted from the blood plasma of Alox15b-KI mice and outbred wildtype controls (n=5 each) and the specified free oxylipins were analyzed by LC-MS (see Materials and Methods).

Table S1. Off-target analysis of our *in vivo* mutagenesis strategy. When we screened the mouse genome with the sequence of our targeting oligonucleotide, we detected 5 potential off-target sequences on different chromosomes. These regions were amplified by genomic PCR and the amplification products were sequenced. In all cases we detected the wildtype sequences.

**Location**	**Number of mismatches**	**Sequencing result**
Chr2 13819347 – 13819369	5	wildtype
Chr2 52611563 – 52611585	3	wildtype
Chr16 14176363 – 14176385	4	wildtype
Chr9 25478640 – 25478662	4	wildtype
Chr15 92238427 – 92238449	5	wildtype

Table S2. Mobile phase gradient used for LC-separation of the plasma oxylipins. The extracted plasma oxylipins were separated by RP-HPLC as described in the Material and Methods section using a gradient of the mobile phase that was made out of two solvent stock solutions: Stock A: water containing 0.05 % acetic acid (low elution power), stock B: 1:1 mixture of methanol : acetonitrile (high elution power).

**Time [min]**	**Solvent stock solution B [%]**
0	5
0.5	56
5.5	61
18.5	87
18.6	98

Table S3. Ionization conditions used in our LC-MS based analysis of the blood plasma oxylipins. The extracted plasma lipids were analyzed by LC-MS as described in the Materials and Method section and the ionization conditions used for MS analysis are specified in this table.

**Parameter**	**Conditions**
Drying gas temperature / flow	105 °C / 16 L/min
Sheath gas temperature / flow	390 °C / 12 L/min
Nebulizer pressure	32 psi
Capillary voltage	- 4300 V
Nozzel voltage	- 1950 V

Table S4. Characteristic mass transitions, collision energies and retention times of the different oy-lipins.

Oxylipin	Precursor Ion	Product ion	MS Res	Collision Energy (V)	Ret Time (min)
10-HDHA	343.2	181.1	Wide	11	16.725
10-HDHA	343.2	153.1	Wide	14	16.725
11-HDHA	343.2	149.1	Wide	12	17.013
11-HDHA	343.2	121.1	Wide	13	17.013
11-HETE	319.2	167.1	Wide	14	16.623
11-HETE	319.2	149.1	Wide	22	16.623
12-HEPE	317.2	179.1	Wide	13	15.01
12-HEPE	317.2	135.1	Wide	13	15.01
12-HETE	319.2	179.2	Wide	13	16.935
12-HETE	319.2	135.1	Wide	14	16.935
12-HeTrE	321.2	181	Wide	20	17.9
12-HeTrE	321.2	209	Wide	22	17.9
13-gamma-HOTrE	293.2	193	Wide	18	14.46
13-HDHA	343.2	221.2	Wide	10	16.546
13-HDHA	343.2	193.2	Wide	12	16.546
13-HODE	295.2	195.2	Wide	18	15.813
13-HODE-d4	299.2	198.1	Wide	18	15.857
13-HOTrE	293.2	195	Wide	18	14.07
13-HOTrE	293.2	223	Wide	20	14.07
14.15-DHET-d11	348.3	207.1	Wide	20	13.031
14.15-DHET-d11	348.3	140.1	Wide	20	13.031
14-HDHA	343.2	205.1	Wide	11	16.734
14-HDHA	343.2	161.1	Wide	13	16.734
15(R)epi-LXA4	351	135	Wide	18	7.186
15(R)epi-LXA4	351	115	Wide	15	7.186
15-HEPE	317.2	219.2	Wide	11	14.648
15-HEPE	317.2	175.1	Wide	13	14.648
15-HETE	319.2	219.2	Wide	11	16.252
15-HETE	319.2	121.1	Wide	16	16.252
15-HETE-d8	327.2	226.2	Wide	11	16.143
15-HETE-d8	327.2	214.2	Wide	14	16.143
15-HETE-d8	327.2	182.1	Wide	14	16.143
17(R)RvD1	375	215	Wide	18	7.227
17(R)RvD1	375	141	Wide	16	7.227
17(R.S)-RvD4	375.3	131	Wide	14	8.51
17(R.S)-RvD4	375.3	101	Wide	21	8.51
17-HDHA	343.2	245.2	Wide	10	16.431
17-HDHA	343.2	201.1	Wide	13	16.431
18-HEPE	317.2	259.2	Wide	9	14.22
18-HEPE	317.2	215.2	Wide	13	14.22
20-HDHA	343.2	241.2	Wide	11	16.059

Oxylipin	Precursor Ion	Product ion	MS Res	Collision Energy (V)	Ret Time (min)
20-HDHA	343.2	227.2	Wide	15	16.059
20-HETE-d6	325.2	295.2	Wide	18	15.045
20-HETE-d6	325.2	281.1	Wide	16	15.045
4-HDHA	343.2	133.1	Wide	14	17.983
4-HDHA	343.2	101	Wide	13	17.983
5(S).15(S)-DiHETE	335.3	173	Wide	14	10.88
5(S).15(S)-DiHETE	335.3	255.1	Wide	18	10.88
5(S).12(S)-DiHETE	335.2	195.1	Wide	16	11.82
5(S).12(S)-DiHETE	335.2	129	Wide	20	11.82
5-HEPE	317.2	201.1	Wide	13	15.48
5-HEPE	317.2	115.1	Wide	11	15.48
5-HETE	319.2	191.2	Wide	14	17.521
5-HETE	319.2	115.1	Wide	14	17.521
5-HeTrE	321.2	115	Wide	14	19.64
5-HeTrE	321.2	259	Wide	15	19.64
6(S)-LXA4	351	135	Wide	19	7.6
6(S)-LXA4	351	115	Wide	13	7.6
7-epi-MaR1	359	297	Wide	13	10.081
7-epi-MaR1	359	177	Wide	16	10.081
7-HDHA	343.2	141.1	Wide	11	17.144
7-HDHA	343.2	113.1	Wide	18	17.144
8.9-EET-d11	330.3	268.2	Wide	12	18.64
8.9-EET-d11	330.3	167.1	Wide	13	18.64
8.9-EET-d11	330.3	159.1	Wide	24	18.64
8-HDHA	343.2	189.1	Wide	11	17.338
8-HDHA	343.2	109	Wide	12	17.338
8-HEPE	317.2	161.1	Wide	17	14.91
8-HEPE	317.2	155.1	Wide	13	14.91
8-HETE	319.2	155.1	Wide	12	16.915
8-HETE	319.2	127.1	Wide	22	16.915
9-HEPE	317.2	167.1	Wide	13	15.201
9-HEPE	317.2	149.1	Wide	13	15.201
9-HETE	319.2	151.1	Wide	13	17.299
9-HETE	319.2	123.1	Wide	15	17.299
9-HODE	295.1	171	Wide	21	15.94
9-HODE	295.1	277	Wide	21	15.94
9-HOTrE	293.2	171	Wide	13	13.88
9-HOTrE	293.2	121	Wide	18	13.88
LTB4	335.2	129	Wide	20	11.45
LTB4	335.2	195.1	Wide	16	11.45
LTB4-6-trans	335.2	195.1	Wide	16	10.54
LTB4-6-trans	335.2	129	Wide	20	10.54
LTB4-6-trans-epi	335.2	195.1	Wide	16	10.89

Oxylipin	Precursor Ion	Product ion	MS Res	Collision Energy (V)	Ret Time (min)
LTB4-6-trans-epi	335.2	129	Wide	20	10.89
LTB4-d4	339.2	197.1	Wide	16	11.59
LTB4-d4	339.2	59	Wide	32	11.59
LXA4	351	135	Wide	19	7.15
LXA4	351	115	Wide	13	7.15
LxA5	349	233	Wide	11	5.656
LxA5	349	115	Wide	18	5.656
LXB4	351	233	Wide	13	6.2
LXB4	351	221	Wide	15	6.2
Maresin 1	359	177	Wide	16	11.38
Maresin 1	359	113	Wide	15	11.38
Maresin 2	359.3	232.2	Wide	15	12.52
Maresin 2	359.3	221.2	Wide	12	12.52
NPD1	359.3	206.1	Wide	15	10.89
NPD1	359.3	153	Wide	15	10.89
NPDx	359.2	206	Wide	16	10.76
NPDx	359.2	153	Wide	16	10.76
PGE2-d4	355.2	275.2	Wide	18	6.185
PGE2-d4	355.2	193.2	Wide	20	6.185
RvD1	375	215	Wide	18	7.073
RvD1	375	141	Wide	16	7.073
RvD2	375	277	Wide	13	6.299
RvD2	375	175	Wide	23	6.299
RvD3	375	147	Wide	22	6.23
RvD3	375	115	Wide	18	6.23
RvD5	359	199	Wide	14	10.98
RvD5	359	141	Wide	13	10.98
RvE1	349	195	Wide	17	4.16
RvE1	349	107	Wide	22	4.16

Table S5. Metabolites quantified in the frame of our plasma lipidome analysis.

Parent PUFA	Metabolite	Detection limit (ng/ml plasma)
9,12-octadecadienoic acid	9-HODE	0.59
	13-HODE	0.30
9,12,15-octadecatrienoic acid	9-HOTrE	0.86
	13-HOTrE	0.86
8,11,14-eicosatrienoic acid	5-HeTrE	0.21
	8-HeTrE	0.92
	12-HeTrE	0.49
	15-HeTrE	0.51
5,8,11,14-eicosatetraenoic acid	5-HETE	0.41
	8-HETE	3.12
	12-HETE	0.67
	15-HETE	0.51
	LTB4	0.28
	5(S),12(S)-DiHETE	0.41
	5(S),15(S)-DiHETE	0.25
	8(S),15(S)-DiHETE	0.35
5,8,11,14,17-eicosapentaenoic acid	5-HEPE	0.41
	8-HEPE	0.20
	12-HEPE	0.46
	15-HEPE	0.72
	18-HEPE	0.52
	RvE1	1.24
4,7,10,13,16,19-docosahexaenoic acid	4-HDHA	0.33
	7-HDHA	0.55
	8-HDHA	1.07
	10-HDHA	0.29
	11-HDHA	0.35
	13-HDHA	1.56
	14-HDHA	0.28
	16-HDHA	0.29
	17-HDHA	0.94
	20-HDHA	0.44
	22-HDHA	0.18
	Maresin 1	0.32
	Maresin 2	0.22
	Maresin 7-epi	0.31
	RvD1	1.84
	RvD1 17(R)	2.50
	RvD2	2.40
	RvD3	1.89
	RvD4 17(R,S)	2.50
	RvD5	1.40
	NPD1	0.17
	NPD-x	0.36

Table S6. Spleen weights of male and female middle-aged Alox15b-KI mice and outbred wildtype controls. Male and female Alox15b-KI mice and outbred wildtype control animals were sacrificed under anesthesia, the spleens were prepared and their weights were determined. The absolute spleen weights of the different individuals of the two different genotypes were compared with the Mann-Whitney U-test.

**Gender**	**Wildtype**		**Alox15b-KI**		U-test
	**Spleen weight (g)**	**n-number**	**Spleen weight (g)**	**n-number**	
male	0.12±0.02	10	0.11±0.02	12	n.s.
female	0.11±0.02	13	0.12±0.01	11	n.s.
